# Group A streptococcal collagen-like protein 1 restricts tumor growth in murine pancreatic adenocarcinoma and inhibits cancer-promoting neutrophil extracellular traps

**DOI:** 10.3389/fimmu.2024.1363962

**Published:** 2024-03-07

**Authors:** Emily A. Henderson, Abby Ivey, Soo Jeon Choi, Stell Santiago, Dudley McNitt, Tracy W. Liu, Slawomir Lukomski, Brian A. Boone

**Affiliations:** ^1^ Department of Microbiology, Immunology and Cell Biology, School of Medicine, West Virginia University, Morgantown, WV, United States; ^2^ West Virginia University Cancer Institute, School of Medicine, West Virginia University, Morgantown, WV, United States; ^3^ Department of Pathology, School of Medicine, West Virginia University, Morgantown, WV, United States; ^4^ Department of Surgery, West Virginia University, Morgantown, WV, United States

**Keywords:** pancreatic cancer, neutrophil extracellular traps, bacteriotherapy, group A *Streptococcus*, myeloperoxidase

## Abstract

**Introduction:**

Pancreatic ductal adenocarcinoma (PDAC) is a lethal cancer associated with an immunosuppressive environment. Neutrophil extracellular traps (NETs) were initially described in the context of infection but have more recently been implicated in contributing to the tolerogenic immune response in PDAC. Thus, NETs are an attractive target for new therapeutic strategies. Group A *Streptococcus* (GAS) has developed defensive strategies to inhibit NETs.

**Methods:**

In the present work, we propose utilizing intra-tumoral GAS injection to stimulate anti-tumor activity by inhibiting cancer-promoting NETs. Mice harboring Panc02 or KPC subcutaneous tumors injected with three different M-type GAS strains. Tumors and spleens were harvested at the endpoint of the experiments to assess bacterial colonization and systemic spread, while sera were analyzed for humoral responses toward the streptococcal antigens, especially the M1 and Scl1 proteins. Role of the streptococcal collagen-like protein 1 (Scl1) in anti-PDAC activity was assessed *in vivo* after intratumoral injection with M1 GAS wild-type, an isogenic mutant strain devoid of Scl1, or a complemented mutant strain with restored scl1 expression. In addition, recombinant Scl1 proteins were tested for NET inhibition using *in vitro* and *ex vivo* assays assessing NET production and myeloperoxidase activity.

**Results:**

Injection of three different M-type GAS strains reduced subcutaneous pancreatic tumor volume compared to control in two different murine PDAC models. Limitation of tumor growth was dependent on Scl1, as isogenic mutant strain devoid of Scl1 did not reduce tumor size. We further show that Scl1 plays a role in localizing GAS to the tumor site, thereby limiting the systemic spread of bacteria and off-target effects. While mice did elicit a humoral immune response to GAS antigens, tested sera were weakly immunogenic toward Scl1 antigen following intra-tumoral treatment with Scl1-expressing GAS. M1 GAS inhibited NET formation when co-cultured with neutrophils while Scl1-devoid mutant strain did not. Recombinant Scl1 protein inhibited NETs *ex vivo* in a dose-dependent manner by suppressing myeloperoxidase activity.

**Discussion:**

Altogether, we demonstrate that intra-tumoral GAS injections reduce PDAC growth, which is facilitated by Scl1, in part through inhibition of cancer promoting NETs. This work offers a novel strategy by which NETs can be targeted through Scl1 protein and potentiates its use as a cancer therapeutic.

## 1 Introduction

Pancreatic adenocarcinoma (PDAC) is one of the most lethal cancers with a five-year survival rate of only 12% (1, 2). Late diagnosis and resistance to current treatment modalities contribute to this poor survival (3, 4). PDAC is an immunologically “cold” tumor, with minimal response to checkpoint immunotherapy (5, 6). Therefore, there is a growing need for the development of strategies to induce an anti-tumor immune response and generate novel cancer therapies in this deadly disease.

William Coley, a bone osteosarcoma surgeon influenced by the work of German physician Wilhelm Busch, performed the first systematic study using streptococcal organisms as a cancer treatment strategy in 1891 leading to the development of Coley’s toxin, a concoction of heat-killed *Streptococcus pyogenes* (group A *Streptococcus*) and *Serratia marcescens*. Though his work fell out of favor with implementation of radiation and chemotherapy, evidence suggests Coley’s intuition of stimulating the immune system to elicit an anti-cancer response is effective (7). Bacteria harbor a variety of structures, toxins and mechanisms that make them attractive candidates as therapeutic agents with some evidence of preclinical efficacy in PDAC (8). Evidence suggests that group A *Streptococcus* (GAS) peptides (9) and superantigens (10) can elicit tumor regression. GAS therapy showed promise in instances of bladder cancer (11), though was less successful in preclinical trial of other cancers (12, 13). Still, GAS utilizes several compounds to evade innate immunity that offer mechanisms by which GAS can stimulate an anti-tumor response. One mechanism is the inhibition of neutrophil extracellular trap (NET) release (14). NETs were initially described as a method for neutrophils to trap and kill bacteria and other infectious agents (15), however NETs are also upregulated in sterile inflammatory diseases including PDAC (16, 17). Recent evidence suggests the significance of NETs in contributing to PDAC tumor growth, progression, metastasis, and promotion of an immunosuppressive tumor environment (18–21). Moreover, NET levels are a prognostic indicator of PDAC patient survival and treatment outcomes (22, 23). GAS has the capacity to evade NET degradation through the activity of streptococcal collagen-like 1 protein which prevents NET release (14, 24), though the use of GAS to limit NETs has yet to be explored in the context of cancer.

Herein, we demonstrate GAS as a potential therapeutic agent in two murine models of PDAC. We also propose the therapeutic significance of streptococcal collagen-like-1 (Scl1) protein as an essential factor in GAS anti-tumor response that supports tumor colonization, and acts as a potent inhibitor of NET activity through reduction of myeloperoxidase activity. These findings have significant implications in the exploration of recombinant Scl1 as a novel therapy to limit PDAC progression.

## 2 Materials and methods

### 2.1 Animals

Age and gender matched C57BL/6 wild-type (WT) mice (8-weeks old) were purchased from the Jackson Laboratory (Bar Harbor, ME, USA). All animal experiments were approved by the Institutional Animal Care and Use Committee of West Virginia University, protocol number 1602000144_R1.5 and 180918204 and complied with the use of experimental animals published by the National Institutes of Health.

### 2.2 Cell lines and cell-culture

Panc02 cells derived from a pancreatic adenocarcinoma model in male C57BL/6 mice were obtained from the National Cancer Institute. KPCY6422c1 cells derived from mouse pancreatic neoplasms were purchased from Kerafast (Kerafast, MA, USA). Panc02 cells were cultured in RPMI 1640 media supplemented with 10% fetal bovine serum and 1% Penicillin-Streptomycin (Hyclone, Logan, UT, USA). KPCY6422c1 cells were cultured with Dulbecco’s Modified Eagle Medium with 10% fetal bovine serum and 1% glutamine. Cells were grown at 37°C in a humidified 5% CO_2_ atmosphere.

### 2.3 Murine subcutaneous tumor model

Age and gender-matched 8-week-old C57BL/6 WT mice were injected with 5x10^5^ Panc02 or KPCY6422c1 cells into the right flank. Once tumors reached 7 mm (10-12 days post tumor implantation), tumors were injected with group A *Streptococcus* as outlined below or with PBS. Caliper measurements of tumors were taken every other day until experimental endpoint. Length (L) and width (W) of tumor were measured and tumor volume in mm^3^ was calculated using equation for spherical ellipsoid (V_T_=0.5*L*W^2^) (25). Tumors and spleens were harvested, weighed, homogenized, and cultured on blood agar (**Supplementary Figure S1**). Tissues were processed for H&E and Gram stains in the West Virginia University Pathology Research Histology Laboratory. Neutrophil index scores from tumor tissues were determined and scored by a blinded pathologist.

### 2.4 Generation of recombinant proteins

Recombinant rScl1 proteins were generated as described previously (26–28). Briefly, rScl1-encoding clones were generated in the *Strep*-tag II cloning, expression, and purification system using the *E. coli* vector pASK-IBA2 designed for periplasmic expression (IBA-GmbH, Geottingen, Germany); rScl1.1 was derived from the Scl1 protein in M1-type strain, rScl1.28 in M28-type strain, and rScl1.41 in M41-type strain. Clones were verified by DNA Sanger sequencing and rScl1 polypeptides by N-terminal sequencing. Bacterial cultures were grown in Luria-Bertani medium (LB broth, Miller) and protein expression was induced with anhydrotetracycline (0.2 μg/mL) for three hours. The periplasmic fraction was collected following treatment with CellLytic B Cell Lysis reagent (Sigma, B7435) and purified on *Strep*-Tactin Sepharose.

rEDA was produced intracellularly using the pQE-30 His tag purification system (Qiagen) in *E. coli* JM-109 (29). *E. coli* was cultured in LB broth supplemented with ampicillin (100 μg/mL) and protein expression was induced with 1 mM isopropyl β-D-thiogalactopyranoside for three hours. Cells were harvested and lysed in lysis buffer (50 mM Tris/HCL, pH 8.0, 50 mM NaCl, 2 mM MgCl_2_, 2% Triton X-100, 10 mM beta-mercaptoethanol, 2 mg lysozyme, 1 mM EDTA-free protease inhibitor cocktail tablet, 1 mM phenylmethylsulfonyl fluoride (PMSF), 100 U DNase I). Supernatant was collected following centrifugation, and rEDA was purified using nickel-nitrilotriacetic acid-agarose resin (Qiagen).

Following purification, rScl1 proteins and rEDA were analyzed by SDS-PAGE (**Supplementary Figure S2**). Proteins were dialyzed in 25 mM HEPES pH 8.0 and stored at -20°C.

### 2.5 Group A *Streptococcus* strain preparation and injection

The M1-type GAS strain MGAS5005 *Δscl1* mutant was generated by allelic replacement mutagenesis as described previously (30). Briefly, *scl1.1* allele was cloned into an *E. coli* vector and coding sequence was replaced with a nonpolar spectinomycin resistance cassette spc2, resulting in pSL134 plasmid suicide in GAS. MGAS5005 cells were electroporated with pSL134 and spectinomycin-resistant colonies were screened by PCR for a single amplification product of mutated size indicating double cross-over event; mutant candidates were then verified by sequencing and loss of Scl1 protein was confirmed by western immunoblotting with specific anti-Scl1.1 Abs.

Complementation of M1 *Δscl1* mutant was performed as previously described (26) using GAS-*E. coli* shuttle plasmid. The DNA fragment encoding *scl1.1* allele was PCR-amplified from genomic DNA of MGAS5005 and cloned into pSB027 generating plasmid pSL620. pSL620 was introduced into MGAS5005 *Δscl1.1* mutant by electroporation and selected on Brain Heart Infusion (BHI) agar with 10 μg/mL chloramphenicol; Scl1.1 expression was confirmed by western immunoblotting as above.

M1 wild type (WT), *Δscl1.1* isogenic mutant, *Δscl1.1::620* complemented mutant, M41 strain MGAS6183, and M28 strain MGAS6143 group A *Streptococcus* were grown overnight at 37°C with 5% CO_2_ on BHI agar solid media supplemented with antibiotics, as needed. Plated bacteria were inoculated into Todd-Hewitt Yeast (THY) broth and incubated until OD_600_ reached ~0.5. Following centrifugation, bacterial cells were washed once with PBS, centrifuged again, and diluted in PBS to designated concentrations (M1; 1x10^5^, 1x10^6^, 1x10^7^; M41 and M28; 1x10^4^, 1x10^5^, 1x10^6^). Indicated inocula of bacteria in 0.1-mL volume were injected into subcutaneous tumors established in C57BL/6 mice. Control mice received intra-tumoral PBS injections at equal volume.

To assess colonization by GAS, tumors collected at the endpoint of experiments were homogenized and plated serially diluted on blood agar; bacteria were enumerated after overnight incubation. In addition, bacteria recovered from tumors injected with *Δscl1.1* isogenic mutant were assessed for mutated genotype after 14 days *in vivo* colonization without antibiotic selection. Serially diluted tumor homogenates were plated on blood agar as above to produce single colonies. Next, 100 streptococcal colonies were re-streaked on blood agar and on BHI agar supplemented with 100 μg/mL of spectinomycin, and incubated overnight (**Supplementary Figure S3A**). In addition, GAS colonies were screened by PCR with primers flanking *scl1* gene and analyzed on 1% agarose gel. The amplicon sizes expected differ between wild-type allele and mutated allele (**Supplementary Figure S3B**).

### 2.6 ELISA and western blot analysis

To assess mouse seroconversion to streptococcal antigens, blood was collected from tumor burdened mice 14 days following GAS injection or PBS controls. For whole-cell ELISA, M1 wild-type bacteria were harvested at OD_600_ ~0.5 and washed as above in PBS. Cells were finally suspended in bicarbonate buffer, pH 9.6; for coating, 1x10^7^ CFU were added into wells of 96-well high binding microplates and incubated at 4°C overnight (31). Wells were washed with tris-buffered saline (TBS)-1% BSA and blocked in this reagent at 37°C for 2 hours. Primary mouse sera were added at a 1:100 dilution in TBS-1% BSA. Anti-M1 and anti-Scl1.1 rabbit antibodies were used as positive controls. Plates were incubated at 37°C for 2 hours. Anti-mouse or anti-rabbit HRP-conjugated secondary antibodies at 1:1000 dilution were added and incubated at room temperature for 1 hour; then, wells were washed and ABST substrate was added, and immunoreactivity was read at 415 nm. To detect anti-Scl1.1 specific seroconversion, ELISA was performed using *Strep*-Tactin coated wells immobilized with rScl1.1 protein, as previously described (32); rScl1.1 construct contains a *strep*-tag II at the C-terminus, which binds to *Strep*-Tactin and thus reflecting the natural orientation of the Scl1 protein as on the GAS bacterial surface.

Western immunoblotting was carried out against GAS cell wall-associated protein fraction prepared from MGAS5005 and separated on 15% SDS–PAGE, as described (30). Log-phase GAS cells were harvested and resuspended in 20% sucrose–10 mM Tris (pH 8.0), 1mM ethylene diamine tetra-acetic acid buffer containing 5 U of mutanolysin,100 μg of lysozyme, and 100 mM PMSF. After digestion at 37°C for 1 h and centrifugation, the supernatants were used for subsequent analyses. The primary antibodies were the same sera (1:500 dil.) used above in whole cell ELISA prepared from mice bled at the endpoint of experiments. The control anti-GAS-antigen antibodies include: (i) anti-M1 protein rabbit Ab (1:2500 dil.) (33) and anti-Scl1.1 affinity purified rabbit Ab (1:1000 dil.) (30). Secondary Abs used were, accordingly, goat anti-mouse IgG(H+L)- or goat anti-rabbit IgG(H+L)-HRP conjugated (Bio-Rad, 1721019), followed by chemiluminescent detection with Clarity Western ECL Substrates (Bio-Rad, 1705060). Western immunoblotting to detect specific anti-rScl1.1 antibodies was performed as described (30). Briefly, rScl1.1 protein was separated by SDS-PAGE, transferred onto a membrane, and probed with mouse sera (1:100 dil.); anti-Scl1.1 rabbit antibodies were used in control well.

### 2.7 Neutrophil extracellular trap assay and quantification

Bone marrow neutrophils were isolated from the femur of euthanized C57BL/6 mice as described previously (16, 34). Bone marrow was collected, and cells were washed with RPMI-10 with 10% FBS and 1% PenStrep. Cells were separated using a density gradient centrifugation with histopaque 1077 and 1099. The layer between 1077 and 1099 was collected and plated onto a 96-well plate at 1.5x10^4^ cells per well in Hank’s Balanced Salt Solution. Neutrophils were incubated for thirty minutes at 37°C to allow for cell attachment. Recombinant Scl1 protein, HEPES, sterile PBS, or whole-cell bacteria were added at the designated concentration and incubated for 5-10 minutes. 50 μM platelet activating factor (PAF) (511075, Sigma Aldrich) was added for activation for 3 hours. Cells were fixed with 4% paraformaldehyde and stained for DNA with Hoechst reagent. NETs were visualized using an EVOS microscope at 10-40x objective. NETs were quantified by collecting the supernatant and measuring cellular free DNA using Quant-iT Picogreen (Invitrogen, Grand Island, NY) according to manufacturer’s protocol. 500,000 U/mL polymyxin B sulfate was added to the recombinant protein for all *in vitro* experiments to account for LPS contamination.

### 2.8 Myeloperoxidase assays and quantification

Recombinant Scl1 proteins were examined for MPO activity using the Myeloperoxidase Inhibitor Screening Assay Kit (700170, Cayman Chemical, Ann Arbor, MI). The proteins were reconstituted in PBS and used in the assay according to the manufacturer’s protocol.

MPO activity was also assessed by luminol bioluminescence imaging of isolated bone marrow neutrophils as described previously (35, 36). Briefly, 1x10^5^ isolated neutrophils were added to a 96-well black walled plate and stained with 100mM luminol (Sigma-Aldrich, MO, USA) followed by stimulation with 500nM phorbol 12-myristate 13-acetate, 99+% (Thermo Scientific Chemicals). Immediately following stimulation, cells were imaged for 60 minutes using the Kino imaging system (Spectral Instruments Imaging, AZ, USA) at 37°C under 5% CO_2_ flow for 12 total acquisitions. Data was analyzed by ROI measurements using Aura software (Spectral Instruments Imaging, AZ, USA) imported into Excel (Microsoft Corp., WA, USA). Data was represented as total flux (photons/second) by averaging triplicate wells and calculating the area under the curve from images taken at 5-60 min.

### 2.9 Statistical analysis

All statistical analysis was performed on GraphPad Prism 10.1.0 (316). Experiments were analyzed for significance using one-way ANOVA with or without repeated measures with multiple comparison *post hoc* tests as indicated in figure legends. Significance is designated as *=p<0.05, **=p<0.01, ***=p<0.001, ****=p<0.0001.

## 3 Results

### 3.1 GAS limits subcutaneous Panc02 and KPC murine PDAC tumor growth

*S. pyogenes* serotype M49 has been shown to elicit anti-tumor growth activity *in vivo* against Panc02 tumors (37, 38). We first sought to substantiate these findings and investigate whether this effect was observable in other M-types or was restricted to M49. We utilized three different strains of *S. pyogenes* (M1, M28, and M41) at three different inocula injected into subcutaneous murine PDAC tumors. One week following injection of 5x10^5^ Panc02 cells into the right flank, male and female mice were randomized to intra-tumoral injection with low, medium, and high inocula of each GAS M type or PBS control at equal volume. Tumor burden was measured on day eight post-infection.

**Figure 1:**
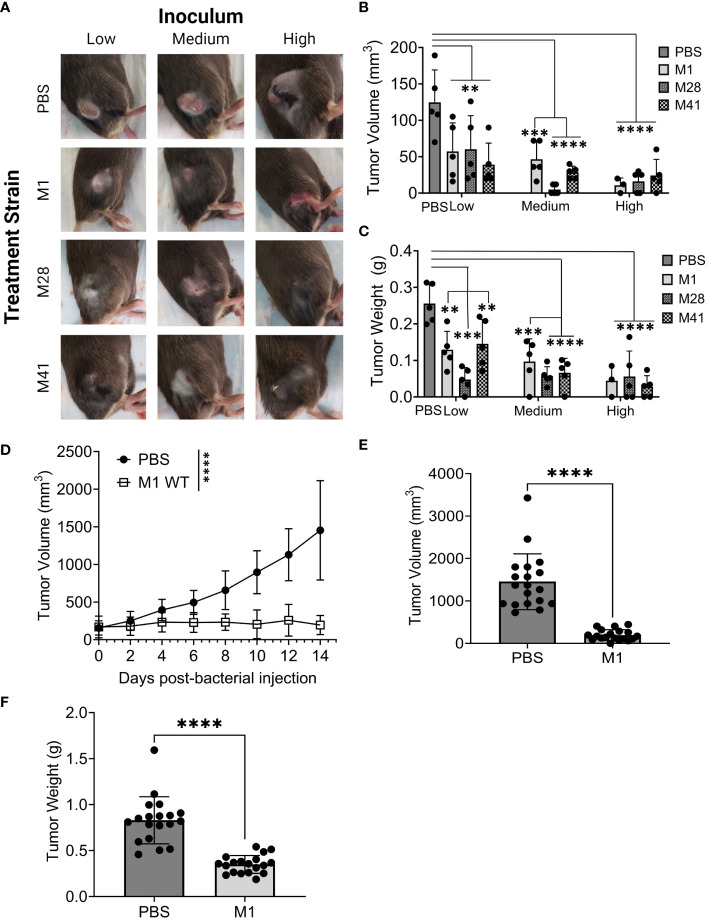
GAS injection limits the growth of subcutaneous murine Panc02 and KPC tumors. **(A)** Representative images of tumors and skin pathology associated with GAS injection. Pre-formed Panc02 tumors were injected intratumorally with GAS strains at low, medium, and high inocula (M1: 10^5^, 10^6^, 10^7^ CFU or M41/M28: 10^6^, 10^7^, 10^8^ CFU) or PBS at equal volume. Data shown were recorded on day 8 post-injection. **(B)** Comparison of tumor volume between experimental groups. Tumor dimensions were measured with a caliper on day 8 post-injection of GAS compared to PBS control. **(C)** Comparison of tumor weight between the groups. Tumors were collected and weighed on day 8 post-injection of GAS or PBS. n=5/group. **(D)** Changes in tumor volume following GAS injection. Measurements were taken by caliper during fourteen days following single-dose treatment of GAS or PBS into murine KPC tumors. Results are representative of two independent experiments. Significance is determined by one-way repeated measures ANOVA. n=19 per group total. **(E)** Tumor volume 14 days post-injection of M1 GAS or PBS. **(F)** Tumor weight at experimental endpoint of M1 GAS treated tumors and PBS control as done in **(B)** Significance determined by student’s *t*-test. **p<0.01, ***p<0.001, ****p<0.0001.

Intra-tumoral injection with all three strains exhibited minimal skin pathology on day 8 (**Figure 1A**), while eliciting significant and comparable reductions in tumor volume and weight using all three strains compared to the PBS-treated tumors (**Figures 1B, C**). Tumor reduction was most pronounced at medium and high inoculum, although there were no significant differences between strains and inoculum sizes. Only two mice inoculated with the high inoculum died from systemic spread of bacteria indicated by the growth of β-hemolytic GAS colonies on blood agar inoculated with blood and spleen homogenates from these mice (**Figure S1**). Overall, we established that GAS of various M-types are capable to reduce Panc02-tumor growth *in vivo*.

The *KRAS* oncogene encodes the protein KRAS involved in regulating cell proliferation and survival. Mutations in *KRAS* can disrupt the switch between active and inactive states thus promoting cancer development (39). Virtually all PDAC tumors harbor *KRAS* mutations and a majority also have mutations in P53, a tumor suppressor protein (3, 40). Therefore, we investigated whether GAS M1 strain had similar anti-tumor effects using KPCY6422c1 cells (abbreviated as KPC), a PDAC tumor lineage that mirrors the genetics of human PDAC (41). We injected 10^6^ CFU, the medium-size inoculum in **Figure 1**, in further experiments to avoid systemic infection with GAS. Mice received a subcutaneous injection of 5x10^5^ KPC cells and were monitored until tumor volumes were within a range of 150-175 mm^3^, around 10 to 12 days, followed by a single intra-tumoral dose of M1 GAS or PBS injection, represented as day 0 post-injection. Tumors were measured every other day for 14 days, e.g., a time, which required to scarify the control PBS-injected mice.

Tumor volume and weight were significantly reduced following treatment with M1 GAS in comparison to PBS control (**Figures 1D–F**). These results indicate that M1 GAS has a persistent therapeutic effect in more aggressive and clinically relevant subcutaneous KPC-PDAC tumors at medium-size inoculum without systemic spread. Based on this data, we established this dosing to be utilized for further studies.

### 3.2 GAS streptococcal collagen-like protein 1 is critical for reduction in PDAC tumor burden and localized tumor colonization

**Figure 2:**
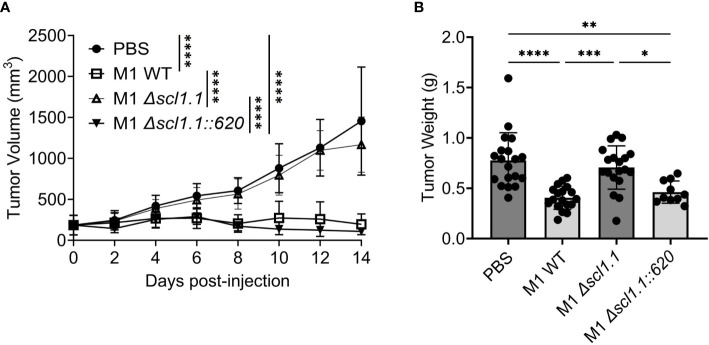
Injection of GAS expressing Scl1 limits the growth of subcutaneous PDAC tumor in mice. **(A)** Changes in tumor volume following GAS injection. Measurements were taken by caliper during fourteen days following single-dose treatment of indicated GAS strains or PBS control in murine KPC tumors. Results are representative of two independent experiments. Significance is determined by one-way repeated measures ANOVA. ****p<0.0001. n=10-20 per group total. **(B)** Tumor weight at experimental endpoint. Mouse tumors treated with indicated strains of GAS or PBS control were extracted and weighed (grams) at day 14. Results are representative of two independent experiments. Each dot represents a single murine tumor. Significance determined by one-way ANOVA with Tukey’s *post-hoc* test. *p<0.05, **p<0.01, ***p<0.001, ****p<0.0001.

Streptococcal collagen-like protein 1 (Scl1) is expressed by majority of GAS strains tested (42–46) and is an important adhesion molecule binding to selected type III repeats of cellular fibronectin (47, 48), a component of the tumoral extracellular matrix also known as oncofetal fibronectin (49). Given that all three-experimental GAS-M-types used express Scl1, which resulted in tumor regression, we sought to explore if Scl1.1 (Scl1 variant in M1-type GAS) was mediating these effects. Following a similar model as above, mice carried subcutaneous KPC-PDAC tumors were administered with a single-dose intra-tumoral injection of either GAS M1 wild-type (WT), an isogenic *Δscl1.1* mutant strain lacking Scl1.1 protein, or a *Δscl1.1::620* mutant strain complemented for Scl1.1 expression. Tumor burden was monitored for fourteen days, then tumors were collected for analyses (**Figure 2**).

Mice treated with the Scl1-expressing WT bacteria exhibited significant decreases in tumor growth over time as well as decreased tumor weights (**Figures 2A, B**). In contrast, tumor volumes in mice injected with the *Δscl1.1* mutant strain showed no such effect and followed the volume pattern of the PBS control group, suggesting that anti-PDAC activity is contingent on Scl1 expression. When Scl1 expression was complemented back into the mutant strain in mice treated with *Δscl1.1::620*, tumor growth was again diminished and similar to injection with the WT strain, validating a critical role for Scl1 in GAS anti-tumor response.

To confirm that the GAS recovered from the tumors treated with the *Δscl1.1* mutant strain did not revert to the wild-type genotype during replication within the tumor, homogenates prepared from two tumors treated with the isogenic *Δscl1.1* mutant strain were serially diluted and plated onto blood agar. 100 colonies from each tumor were streaked in duplicate onto blood agar and BHI supplemented with spectinomycin (*Δscl1.1* mutant was generated using a spectinomycin-resistant cassette) (**Supplementary Figure S3A**) - all 200 colonies grew on the selective medium, thereby, verifying *Δscl1.1*-mutant phenotype. 10 colonies from tumor 1 and 7 colonies from tumor 2 were arbitrarily selected and analyzed by colony PCR for the size of the amplicon corresponding to either to the *scl1.1* wild-type or the *Δscl1.1* mutant allele. All selected colonies produced the same single amplicons corresponding in size to the *Δscl1.1* mutant but not the wild-type allele (**Supplementary Figure S3B**). Taken together, the *Δscl1.1* mutant strain recovered from tumor homogenates retains the mutant genotype and validates the anti-tumor effects seen with GAS-expressing Scl1 during tumor colonization.

**Figure 3:**
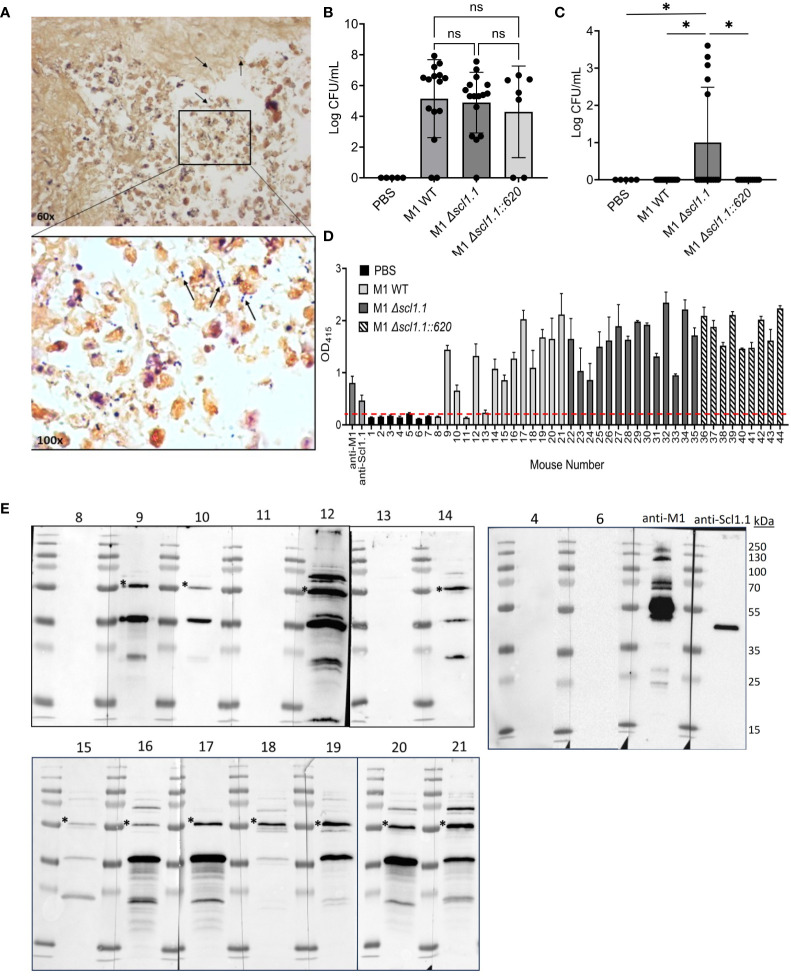
Scl1 promotes localized tumor colonization by GAS without eliciting strong humoral response. **(A)** Histopathology of GAS-treated KPC tumors. Representative Gram-stained image from the KPC tumor treated with GAS (600x magnification). Arrows indicate GAS (1000x magnification) viewed under immersion oil. **(B)** Detection of GAS in KPC tumors. Homogenates were prepared from tumors collected from mice treated with GAS or PBS and serial dilutions were cultured on blood agar, then enumerated. **(C)** Detection of GAS in spleen homogenates. Spleens were processed and analyzed for GAS presence as above (panel B) for tumor samples. Results are from 2-3 independent experiments. Significance is determined by one-way ANOVA. *p<0.05. **(D)** Mouse seropositivity for streptococcal cells developed during tumor colonization by whole-cell ELISA. Mouse sera (1:100 dil.) were from blood collected 14 days after single-dose treatment with either GAS M1 WT (Nos. 8-21; light gray bars), M1 *Δscl1.1* (Nos. 22-35; dark gray bars), M1 *Δscl1.1::620* (Nos. 36-44; striped bars) or PBS (Nos. 1-7; black bars) and tested for immunodetection of GAS surface antigens; specific anti-M1 (1:1000 dil.) and anti-Scl1.1 (1:1000 dil.) Abs were used as positive controls. Each bar represents the average of three technical replicates plus SD. Threshold is indicated by dashed line and was determined by averaging reading of PBS-treated mice at OD _415 nm_. **(E)** Mouse seroconversion to GAS antigens developed during tumor colonization by western blot analysis. The same mouse sera (1:500 dil.) tested in D were reacted against the blots of the M1-GAS cell-wall protein fraction resolved by 15% SDS-PAGE. Asterisk indicates presumed M1-protein immunoreactive band. Specific anti-M1 (1:5000 dil.) and anti-Scl1.1 (1:1000 dil.) Abs were used as positive controls. ns, not statistically significant.

Based on known specific Scl1 interactions with oncofetal fibronectin in the tumor extracellular matrix (47), we hypothesized that Scl1 facilitates a focused nidus of GAS infection, localizing bacteria to the tumor site. Tumor-bearing mice were injected with either GAS M1 WT, an isogenic *Δscl1.1* mutant, or a *Δscl1.1::*620 complemented mutant. Fourteen days post-injection, tumors and spleens were harvested for histopathological analysis and bacterial culture on blood agar (**Figure 3**). Tumors were locally colonized by GAS strains, as evidenced on Gram-stained histograms and on significant recovery of GAS in cultures from tumor homogenates plated on blood agar at the 14-day endpoint post-injection (**Figures 3A, B**). There were no statistically significant differences in tumor colonization levels (CFU per mL) between GAS isogenic strains. However, 5 out of 19 (26%) spleen homogenates recovered from mice treated with the isogenic *Δscl1.1* mutant strain yielded bacterial growth, while no growth occurred from the spleens of mice treated with the WT and *Δscl1.1::620* complemented strains (**Figure 3C**). These data suggest that Scl1 plays a role in localizing GAS to the tumor microenvironment (TME), thereby limiting systemic infection with bacteria.

Consequently, we next asked whether 14-day-long tumor colonization was sufficient to induce host seroconversion toward M1 bacteria, which would demonstrate immune response to the bacterial injection. Mouse sera (1:100 dil.) collected at the endpoint of the experiment (day 14) following a single-dose injection of GAS or PBS control were screened for anti-M1 GAS immunoreactivity by whole-cell ELISA (**Figure 3D**; **Supplementary Figure S4A**). 11 out of 14 (79%) mouse sera collected from mice treated with wild-type bacteria (mice Nos. 8-21) – and none of 7 PBS controls (Nos. 1-7) - exhibited seroconversion to whole bacteria. Similarly, all sera collected from 14 mice treated with the *Δscl1.1* mutant strain (Nos. 22-35) and 9 with *Δscl1.1::620* complemented strain (Nos. 36-44) exhibited seroconversion to whole bacteria. However, there were no significant differences in responses to whole-cell bacteria between GAS-treated groups (**Supplementary Figure S4A**). All 14 sera Nos. 8-21 (1:500 dil.) from mice treated with the wild-type GAS plus 2 sera from PBS controls (Nos. 4 and 6) (**Figure 3E**), as well as sera from mice treated with *Δscl1.1* mutant and with *Δscl1.1::620* complemented strains (8 each) (**Supplementary Figure S4B**), were subsequently assessed for antibody reactivity against GAS WT cell-wall protein fraction by western immunoblotting. The same 11 mouse sera from the wild-type group, previously indicating anti-GAS seropositivity in whole-cell ELISA, consistently produced three major distinct immunoreactive bands of about 55 kDa, 35 kDa, and 20 kDa, with the 55-kDa band co-migrating with M1-protein control. As expected, PBS control sera and the remaining 3 sera from GAS-injected mice that were negative in whole-cell ELISA did not show immunoreactive bands against GAS cell wall antigen preparations. Sera from mice treated with the *Δscl1.1* mutant strain and *Δscl1.1::620* complemented strain also produced immunoreactive bands; however, band patterns were more variable, although nearly all showed a band co-migrating with M1-protein control. The lack of clear seroconversion to Scl1 antigen in sera from the wild-type group was surprising given that Scl1.1 expression, which is controlled by the positive GAS transcriptional regulator Mga (30, 50), is highly transcribed in M1-GAS (44) and has previously demonstrated to elicit humoral responses in both humans and mice (32). Therefore, we hypothesized that mouse sera may not react strongly to the preparation of the cell-wall protein fraction.

To test this hypothesis, representative mouse sera obtained from GAS-WT- and PBS-treated groups were analyzed (1:100 dil.) against purified rScl1.1 protein, instead of the cell-wall protein fraction (**Supplementary Figure S5**). In an ELISA assay, sera from several mice demonstrated immunogenic responses to Scl1.1 antigen, while the PBS mice did not (**Supplementary Figure S5A**). Sera (1:100 dil.) showing positivity in rScl1.1-coated wells, as well as combined control PBS sera, were subsequently analyzed at this concentration (higher than 1:500 dil. in **Figure 3E**) by western immunoblotting following rScl1.1 separation by SDS-PAGE. Under these experimental conditions we could detect bands immunoreactive for rScl1.1 protein (**Supplementary Figure S5B**), in-line with responses seen in the ELISA. Altogether, our data indicate that mice elicit humoral responses against M1-GAS antigens *in vivo*, which includes a relatively weak response against Scl1.1 antigen expressed during tumor colonization.

### 3.3 Intratumoral GAS infection is associated with neutrophil infiltration and Scl1-mediated inhibition of neutrophil extracellular trap formation

**Figure 4:**
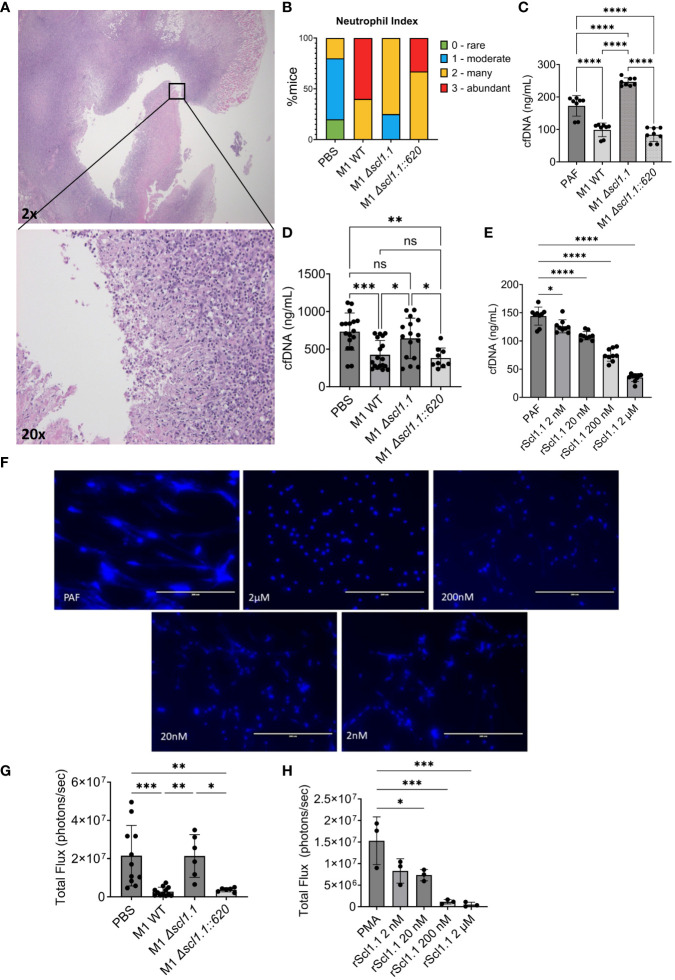
GAS-Scl1.1 impacts intratumoral neutrophil density *in vivo* while rScl1.1 inhibits neutrophil extracellular trap formation *in vitro*. **(A)** Neutrophil infiltration into M1 GAS treated KPC tumors. Representative H&E stains from tumor specimen at low magnification (top image, 20x total magnification) shows neutrophil infiltration at higher magnification (bottom image, 200x total magnification). **(B)** Neutrophil index in KPC tumors. PMN infiltration was scored in tumors from experimental groups harvested 14 days post-injection of a single dose of Scl1 GAS compared to PBS controls **(C)** NET inhibition induced by Scl1.1-expressing GAS. *Ex vivo* NET assay measuring cell-free DNA (cfDNA) concentration following co-incubation of neutrophils and designated bacterial strains quantified by Picogreen assay. Results are representative of three-independent experiments (eight technical replicates). Significance determined by one-way ANOVA with multiple comparison test. **(D)** NET inhibition induced by Scl1.1-expressing GAS *in vivo*. NET assay measuring cfDNA concentration in serum collected from mice treated with indicated strains of GAS or PBS control. Each point represents an individual mouse from two-independent experiments (n=9-18 total/group). Significance determined by one-way ANOVA with multiple comparisons test. **(E)** Quantification of NET inhibition by recombinant proteins. cfDNA concentration measured via Picogreen assay of neutrophils co-incubated with indicated concentrations of rScl1.1 followed by stimulation with PAF. Results are representative of two to three independent experiments (eight to nine technical replicates total. Significance determined by one-way ANOVA with multiple comparison test. **(F)** NET inhibition induced by rScl1.1 *in vitro.* Representative images of neutrophils co-incubated with or without rScl1.1 and stimulated with PAF. NETs are indicated by Hoechst staining. **(G)** MPO activity of neutrophils isolated from treated, tumor-bearing mice. Total flux as measurement of MPO activity of BMNs isolated from mice 14 days after injection of a single dose GAS treatment or PBS control, demonstrating that Scl1 mediates MPO activity from isolated neutrophils. Results are representative of two independent experiments (n=6 total). Significance is determined by one-way ANOVA and multiple comparison test. **(H)** MPO activity of neutrophils treated with rScl1.1. Total flux as measurement of MPO activity of murine BMNs co-incubated with rScl1.1 at indicated concentrations, showing a dose dependent inhibition of MPO. Results are representative of three independent experiments (9 technical replicates total). Each point represents the average area under curve of three technical replicates. Significance is determined by one-way ANOVA and multiple comparison test. *p<0.05, **p<0.01, ***p<0.001, ****p<0.0001. ns, not statistically significant.

Given the profound Scl1-dependent anti-tumor effects of GAS bacteria in murine PDAC, we began to consider potential immunostimulatory mechanisms that could be driving this treatment response. We observed that tumors treated with M1 WT and *Δscl1.1::620* complemented strains had higher neutrophil inflammatory indices of 2-3, scored by a blinded pathologist, compared to indices 1-2 recorded for the *Δscl1.1* mutant strain and PBS control (**Figures 4A, B**). These group differences, e.g., Scl1.1-positive (WT and complemented strains) and Scl1.1-negative (PBS and Scl1.1-devoid mutant), may indicate that higher neutrophil indices observed in Scl1-positive groups could result from either higher PMN infiltration or the possibility of fewer NETs in these experimental groups (since NETs are a form of neutrophil cell death). Previous studies demonstrated that NETs can enhance pancreatic tumor growth (21) and promote metastasis (18). We hypothesized that Scl1.1 may be working, in part, through a neutrophil mediated mechanism to exert its anti-PDAC effects. Previously, Scl1.1 was found to reduce NET formation and killing *in vitro* (24). Therefore, we sought to further explore the role of Scl1.1 in cancer-promoting NETs. Bone marrow neutrophils were isolated and incubated with whole cell bacteria (M1 wild-type, an isogenic Scl1.1-devoid mutant Δ*scl1.1*, and a complemented mutant strain Δ*scl1.1::620*) followed by stimulation with the NET inducer platelet activating factor (PAF). NET production was quantified as a measurement of cell-free DNA (cfDNA) released into the supernatant. Neutrophils co-incubated with the Scl1.1-expressing bacteria produced significantly less cell-free DNA as compared to the Scl1.1-lacking mutant and PAF-only control, consistent with reduced NET formation (**Figure 4C**).

We sought to determine the efficacy of treatment with Scl1.1-expressing bacteria on circulating NETs *in vivo*. Sera were prepared from blood collected from mice 14 days following treatment with a single-dose intra-tumoral injection of M1 wild-type, an isogenic Δ*scl1.1* mutant, the complemented Δ*scl1.1::620* mutant strain, or PBS control. Sera (1:10) were assessed for cfDNA quantification, a validated marker of *in vivo* NET formation in murine PDAC (17). Mice treated with the wild-type and complemented strains had significantly reduced cfDNA levels in comparison to PBS and Scl1.1-lacking mutant, suggesting reduced circulating NETs (**Figure 4D**).

Because GAS has multiple antigenic influences and mechanisms with the potential to inhibit NET formation (14, 51–53), we deemed it necessary to confirm Scl1 as the major driver of NET evasion by employing recombinant Scl1 (rScl1) proteins (**Supplementary Figure S2**) derived from GAS M1 (rScl1.1), M28 (rScl1.28), and M41 (rScl1.41) strains that inhibited the growth of Panc02 tumors (**Figure 1**). Bone marrow neutrophils were isolated and incubated with indicated rScl1 proteins or rEDA control recombinant protein. NET release was assessed by measuring the cfDNA and microscopic imaging following fluorescent staining of DNA with Hoechst reagent. Co-incubation of neutrophils with all three distinct rScl1 protein variants significantly inhibited NET formation (**Supplementary Figure S6**). rScl1.1 had more potent NET reduction in comparison to other rScl1 proteins tested. Moreover, rScl1.1 inhibited NETs in a concentration-dependent manner reaching ~70% inhibition at 2 μM (**Figures 4E, F**). Overall, we demonstrate for the first time that recombinant Scl1 can inhibit NET formation by the same capability as whole cell bacteria, suggesting strong potential as a useful therapeutic approach for NET inhibition.

We next sought to determine the mechanism of Scl1.1 NET inhibition. Myeloperoxidase (MPO) activity is a critical step in NETosis whereby MPO stored in the azurophilic granules of neutrophils interacts with neutrophil elastase to induce chromatin decondensation and subsequent NET release (54). Previous studies demonstrated that Scl1.1 expressed on M1-type GAS suppresses NET formation through inhibition of MPO activity *in vitro* (24). However, at the current time of publication, these results have not been established in cancer models. Here, we hypothesized that treatment with Scl1-expressing GAS limits MPO activity and subsequent NET formation from neutrophils harvested from our murine model using a novel *ex vivo* bioluminescence assay. Bone marrow neutrophils isolated from mice treated with GAS M1-wild type, the isogenic Scl1.1-negative mutant and a complemented Scl1.1-positive strain, or PBS control were stained with the bioluminescence compound luminol, which measures MPO activity (55) and stimulated with phorbol myristate acetate (PMA), a compound known to induce activation of neutrophil NADPH-oxidase and trigger degranulation (55). Neutrophils isolated from mice treated with the Scl1.1 expressing wild-type and complemented strains exhibited significantly less MPO activity compared to neutrophils from PBS controls or treated with the Scl1.1-negative mutant strain (**Figure 4G**), validating that Scl1.1 reduces MPO activity.

We next assessed if rScl1.1 protein could elicit similar reductions in MPO activity as seen in our murine model using live bacteria. As described, bone marrow neutrophils were isolated and co-incubated with rScl1.1 at different concentrations followed by luminol staining and PMA stimulation. MPO activity was significantly reduced in neutrophils treated with rScl1.1 in a concentration-dependent manner (**Figure 4H**). To validate these results, we assessed rScl1.1 in a commercially available MPO inhibitor screening assay, demonstrating that rScl1.1 inhibited MPO production even at low concentrations of protein (**Supplementary Figure S7**). Taken together, these results indicate a potential mechanism by which Scl1 inhibits NET formation by hindering MPO activity and offers therapeutic value to rScl1.1.

## 4 Discussion

PDAC is an aggressive cancer characterized by low survival due to failure of early detection and marked treatment resistance. Despite recent advances in cytotoxic regimens, current therapeutic interventions offer minimal treatment response or survival benefit (56), which necessitates exploration into alternative strategies. William Coley established the significance of GAS in treating bone and soft-tissue sarcomas. However, discrepancies in Coley’s work and the development of radiation and chemotherapy brought disfavor towards the use of Coley’s Toxin (7). In the present work, we elucidate the significance of GAS as a potent anti-cancer agent in PDAC through inhibition of cancer promoting NETosis and re-examine the therapeutic value of bacteriotherapy using GAS.

GAS and other bacteria are attractive candidates for cancer therapy in their capabilities to be recognized by immune cells even in immunosuppressive tumor environments (8). This recognition has the capacity to switch this “immunologically cold” environment to a more immunostimulatory one (57). As demonstrated by Maletzki et al. (2008) using Panc02 subcutaneous model, a single intra-tumoral injection of M49-type GAS can mediate anti-tumor effects through direct tumor lysis and stimulation of anti-tumor immunity by active infection. In the present study, injection with M1, M28, and M41 GAS each significantly reduced Panc02 tumor growth. Moreover, a subsequent use of the M1 GAS led to tumor reduction of a more aggressive and clinically relevant cell lineage such as the KPCY6422c1 line, which solidifies this concept and offers greater potential for clinical translation.

A hallmark of PDAC is an immunosuppressive tumor micro-environment that renders tumors resistant to current immunotherapies (58, 59). Existing literature demonstrates the contribution of NETs to this immunologically cold state (60, 61) and their enhancement to cancer progression (17, 62, 63) and reduced patient survival (23, 64). Thus, NETs are encouraging targets for further cancer therapies. The current work strengthens existing data generated in a non-cancer inflammatory model to suggest that Scl1 on GAS cells is a potent NET inhibitor through reduction in MPO activity (14, 24). Based on these findings, we tested three recombinant Scl1 proteins - derived from GAS M types above inhibiting PDAC growth - rScl1.1, rScl.28, and rScl1.41 for potential NET inhibition. Neutrophils co-incubated with those rScl1 constructs had a decreased propensity to form NETs, as indicated by reduced cell-free DNA concentrations similar to the effects obtained with Scl1-expressing live bacteria. Further, rScl1.1 inhibited MPO activity indicated by an *in vitro* luminol assay. Taken together these results suggest that rScl1 reagents function in a comparable manner as their native proteins expressed in live bacteria. This original finding potentiates rScl1 as a conceivable PDAC therapeutic agent to combat concerns accompanying live and whole bacterial therapy and represents an innovative advance in the field, as recombinant Scl1 has not been previously tested to inhibit cancer-associated NET formation.

Our previous work reported that Scl1 selectively binds to cancer-associated fibroblast (CAF)-deposited oncofetal fibronectin (47, 48, 65), which can modulate the tumor extracellular matrix microenvironment (49). Here, we observed that injection of Scl1.1-expressing GAS was associated with tumor-localized nidus of infection persisting for an extended time, whereas Scl1.1-lacking isogenic mutant had greater systemic bacterial dissemination. Despite these encouraging results in preclinical models in the current study, injection of live GAS may not be a translatable long-term strategy in humans given the potential for pathogenic infection or would require extensive investigations to generate and test attenuated strains still retaining anti-PDAC activity. Our findings implicated Scl1 in the therapeutic efficacy of GAS, with Scl1-devoid mutant strain demonstrating no significant treatment response comparable to PBS control. Taken together with the evidence demonstrating robust neutrophil infiltration at sites of tumor colonization by GAS, these results potentiate the capacity of Scl1 to counteract the immunotolerant environment and function as an anti-cancer therapeutic. Given the observed activity of Scl1 from our previous work (47, 65), the anti-tumor effects observed by Scl1 are likely to be multifactorial. Therefore, further analysis isolating the role of NET inhibition and elucidating specific immune infiltrates and inflammatory queues will offer a more mechanistic approach to characterizing immunomodulatory responses within the tumor microenvironment in response to GAS bacteriotherapy.

Of interest when considering future therapeutic strategies, we observed host immunity to GAS antigens, thus, increasing the likelihood of resolution of inflammation and the need for additional treatment administration to induce a durable response. Repeat treatment within 3 weeks is well in line with currently utilized cytotoxic treatment regimens and is clinically realistic. Experimental treatments utilizing other bacteria, such as *Listeria* (66), show promise and require multiple rounds of injection as well. However, this observation expresses a need to understand specific host-immune responses to bacterial antigens to identify proper dosing. Previous work demonstrating antigenicity and humoral responses against Scl1.1 (32) in addition to the capacity of M1-type GAS to strongly transcribe the *scl1.1* gene *in vitro* (44–46) suggests that injection with M1-type strain could induce robust immune responses to both M1 and Scl1.1 proteins, although this concept was not explored in a tumoral setting. Herein, we report that mice seroconvert to live bacteria, as evidenced by the positive immunoreactivity detected in sera prepared from mouse blood collected 14 days post-GAS injection via whole-cell ELISA and western immunoblotting. While this finding is not surprising given the prominent antigenicity of M proteins (33, 67) and Scl1.1 (32), it poses potential challenges to future studies examining the efficacy of repeated GAS therapy. However, highly immunogenic antigens are beneficial in the context of vaccines (68). Therefore, we cannot exclude the possibility of utilizing these proteins in cancer vaccine development to elicit potent antitumor immunity. We expected Scl1.1 being expressed *in vivo*, as a Mga-regulated protein (30, 50). Surprisingly, western blot analysis did not detect immunoreactive band corresponding to Scl1.1 control in serum (1:500 dil.) sample set during initial testing against M1-GAS cell-wall preparation. However, subsequent analysis utilizing a purified rScl1.1 protein and higher concentration of mouse immune sera (1:100 dil.) demonstrated that mice do seroconvert to Scl1.1 antigen, though this response seemed weakly immunogenic under these experimental conditions. The tumor microenvironment is highly complex and immunosuppressive, and the anti-tumor effects demonstrated herein are suggested to be Scl1-mediated. Therefore, identifying the complex immunological responses within the tumor microenvironment is necessary to determine immunotolerance at later intervals of treatment. Given the lack of seroconversion demonstrated in these early studies, future studies investigating the therapeutic efficacy of Scl1.1 can be conducted utilizing rScl1.1 or engineered non-pathogenic or attenuated bacteria expressing Scl1.1, as opposed to live M1-WT GAS.

In conclusion, we demonstrate a profound anti-tumor effect of several GAS strains, validated in two PDAC model cell lines. We also identify a novel role for Scl1.1 in PDAC therapy and provide insight on future applications utilizing rScl1.1 to target cancer-promoting NETs, which is the subject of ongoing research. We also address limitations of current and future bacteriotherapies through analysis of seropositivity. Taken together, Scl1.1 could be a beneficial therapeutic approach in PDAC that warrants additional studies.

## Data availability statement

The original contributions presented in the study are included in the article/**Supplementary Material**, further inquiries can be directed to the corresponding author/s.

## Ethics statement

The animal study was approved by Institutional Animal Care and Use Committee of West Virginia University. The study was conducted in accordance with the local legislation and institutional requirements.

## Author contributions

EAH: Conceptualization, Investigation, Writing – original draft, Writing – review & editing. AI: Conceptualization, Investigation, Writing – original draft, Writing – review & editing. SJC: Investigation, Writing – review & editing. SS: Visualization, Writing – review & editing, Conceptualization. DHM: Methodology, Writing – review & editing. TL: Methodology, Writing – original draft, Writing – review & editing, Conceptualization. SL: Conceptualization, Funding acquisition, Investigation, Supervision, Writing – original draft, Writing – review & editing. BB: Conceptualization, Funding acquisition, Investigation, Supervision, Writing – original draft, Writing – review & editing. 
