# Supplementary material for: Group A streptococcal collagen-like protein 1 restricts tumor growth in murine pancreatic adenocarcinoma and inhibits cancer-promoting neutrophil extracellular traps
Source: Front Immunol. 2024 Mar 7;15:1363962. doi: 10.3389/fimmu.2024.1363962 (PMC10955053; doi:10.3389/fimmu.2024.1363962)
Supplement: Supplementary file 1 [file DataSheet_1.docx]

Supplementary Material

# Supplementary Figures


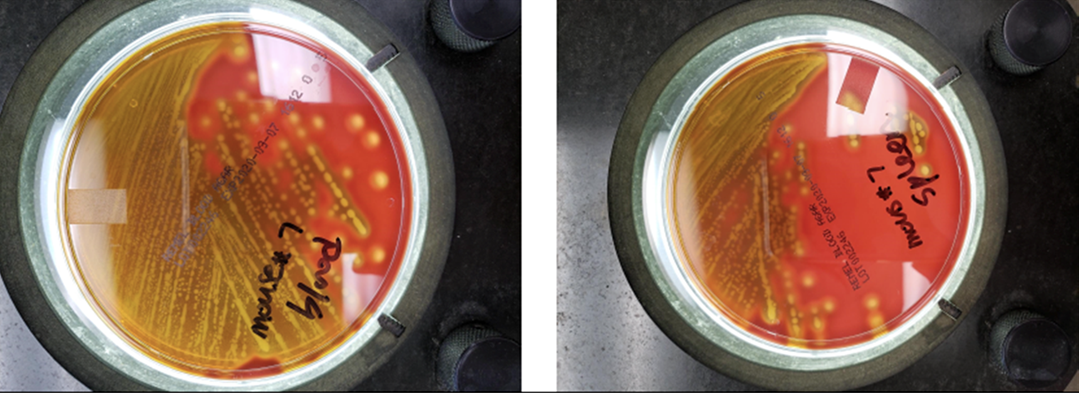


**Supplementary Figure 1. Detections of M1 GAS in blood and organs.** Images of blood and spleen homogenate from representative infected mouse were cultured on blood agar yielding β-hemolytic colonies confirming GAS presence in mouse #7.

**
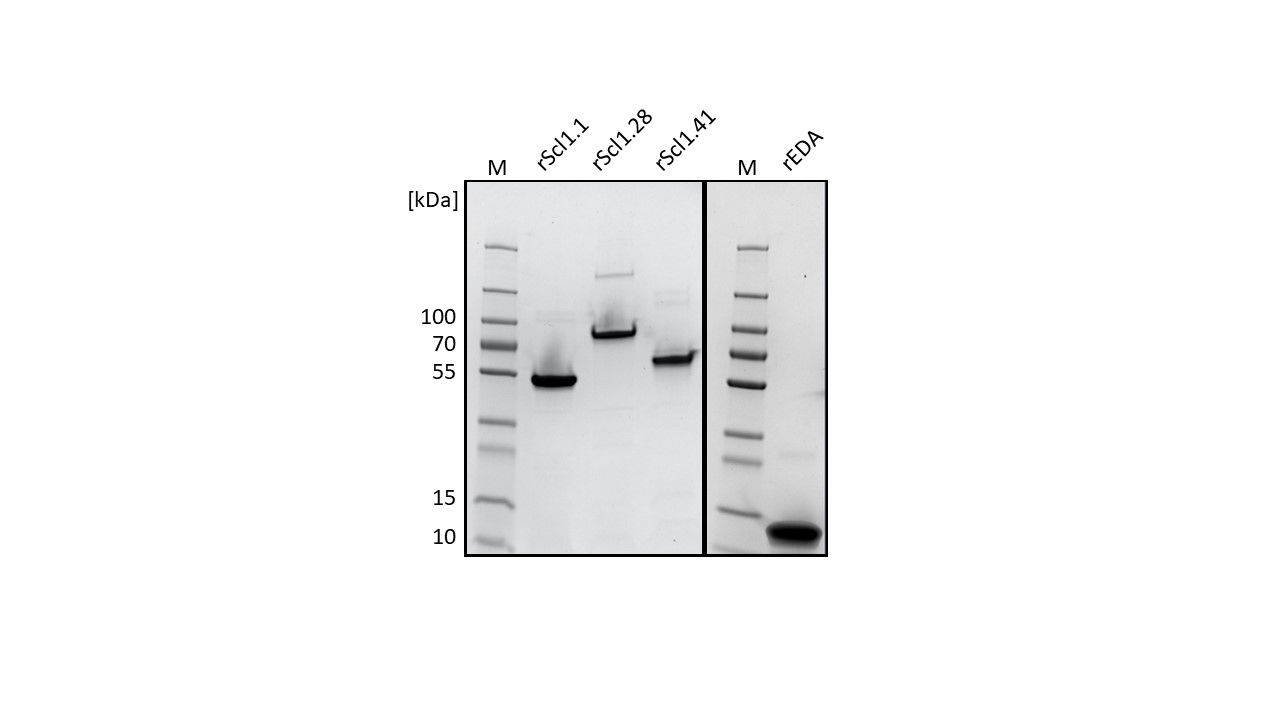
**

**Supplementary Figure 2. Purified recombinant proteins.** 4-20% SDS-PAGE confirming purity and integrity of rScl1 and rEDA preparations. rScl1.1 corresponds to Scl1 in M1-type strain, rScl1.28 in M28-type strain, rScl1.41 in M41-type strain. M, molecular mass standard in kDa.


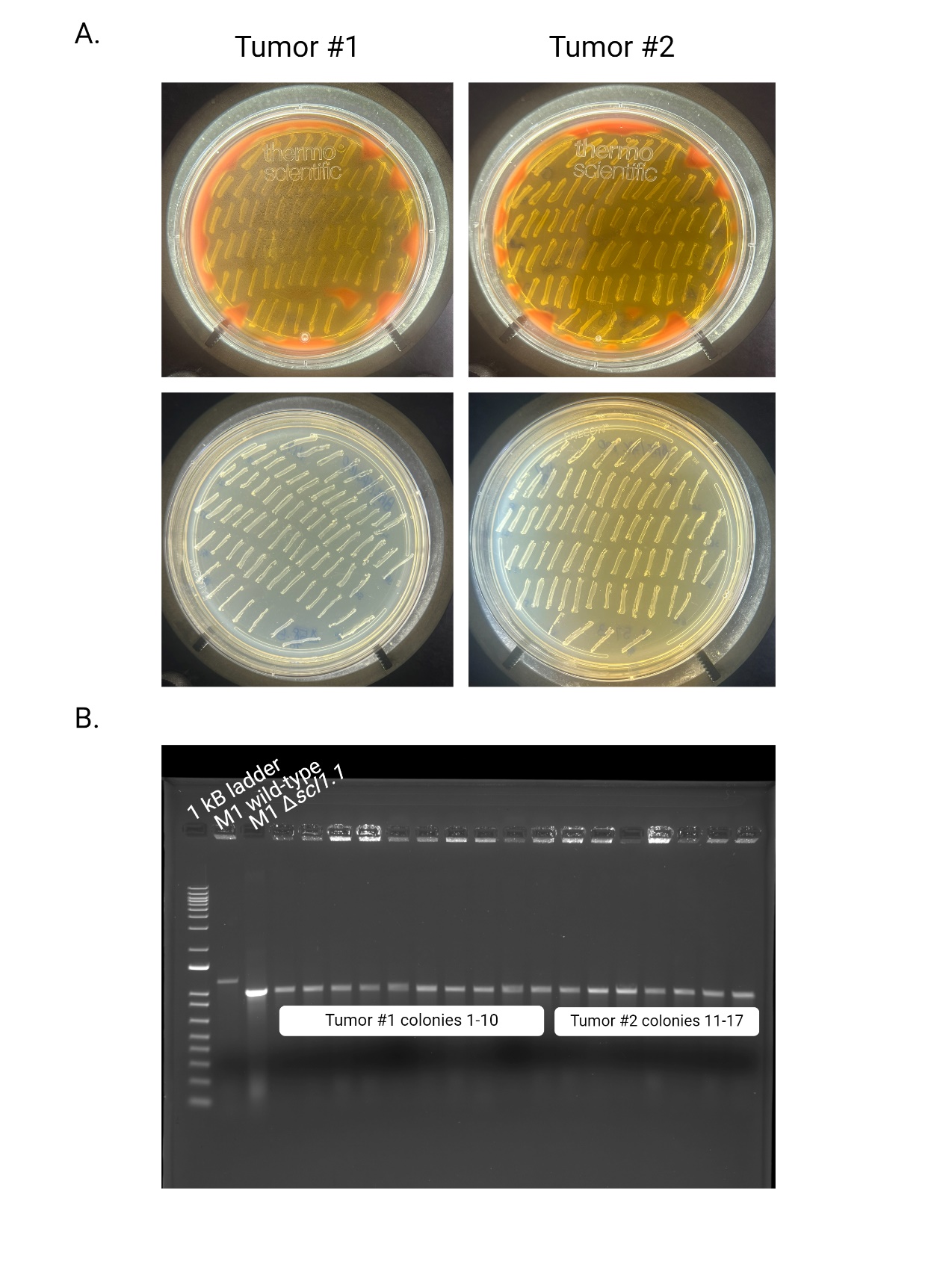


**Supplementary Figure 3. GAS from tumors treated with the *Δscl1.1* mutant strain retains the mutant phenotype.** (A) Representative photographs of blood agar (top) and BHI agar supplemented with spectinomycin (bottom) streaked in duplicate with colonies isolated from tumor homogenates injected with the *Δscl1.1* mutant. plated initially on blood agar, then, 100 colonies recovered from a single tumor were re-streaked on two media, as above. (B) Identification of an amplicon corresponding to the *Δscl1.1* mutant strain but not wild-type strain from recovered bacteria by colony PCR. M1 wild-type and M1 *Δscl1.1* were used as expected size markers; 1-kb DNA ladder is also shown.


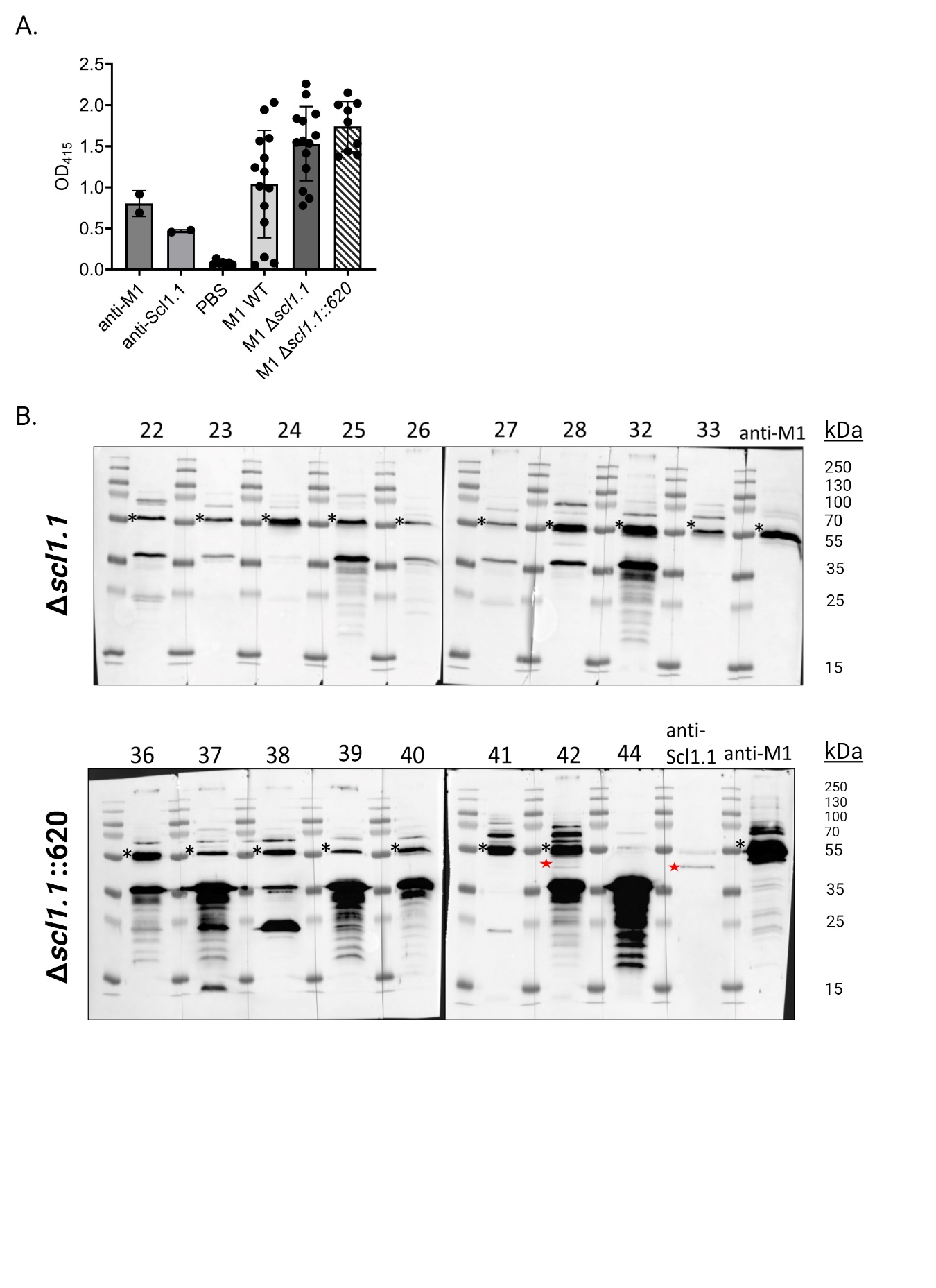


**Supplementary Figure 4. Mouse seroconversion to GAS antigens.** (A) Seropositivity towards GAS antigens developed during tumor colonization from sera collected from mice treated with M1 wild-type, the isogenic *Δscl1.1* mutant strain, the *Δscl1.1::620* strain, or PBS control. Each point is representative of the average of three technical replicates. Error bars indicate SD. (B) Seropositivity towards GAS antigens developed during tumor colonization by the isogenic *Δscl1.1* mutant strain (top) and *Δscl1.1::620* strain (bottom) analyzed by western immunoblotting. Numbers correspond to mouse numbers as in main figure 3D. Asterisks are indicative of presumed M1-immunoreactive band. Red stars are indicative of Scl1.1-immunoreactive band.

**A.**

**
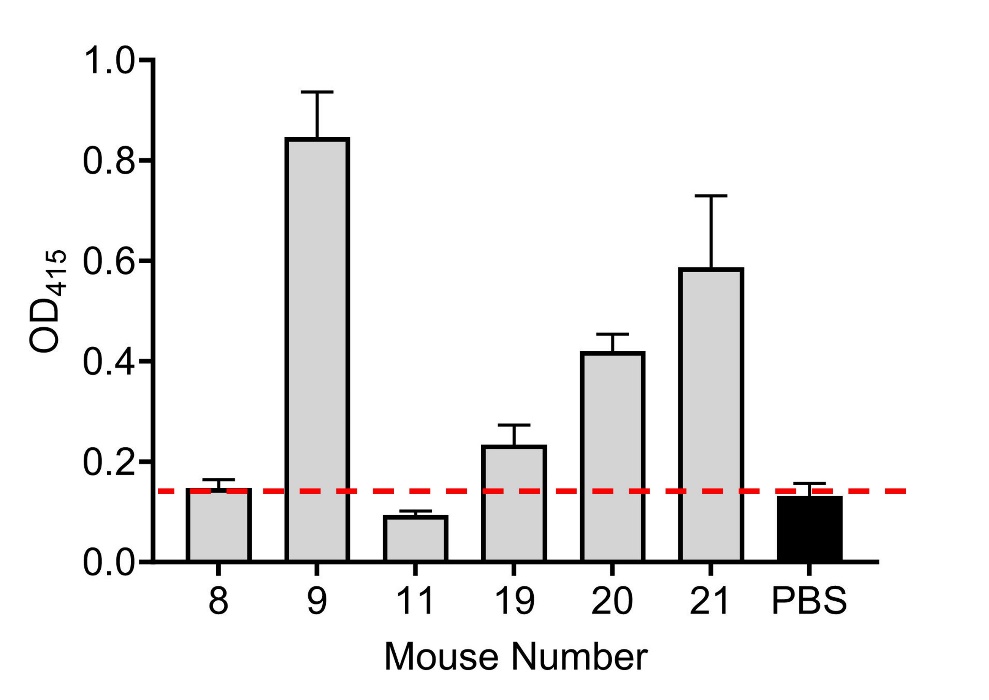
**

**B.**

**
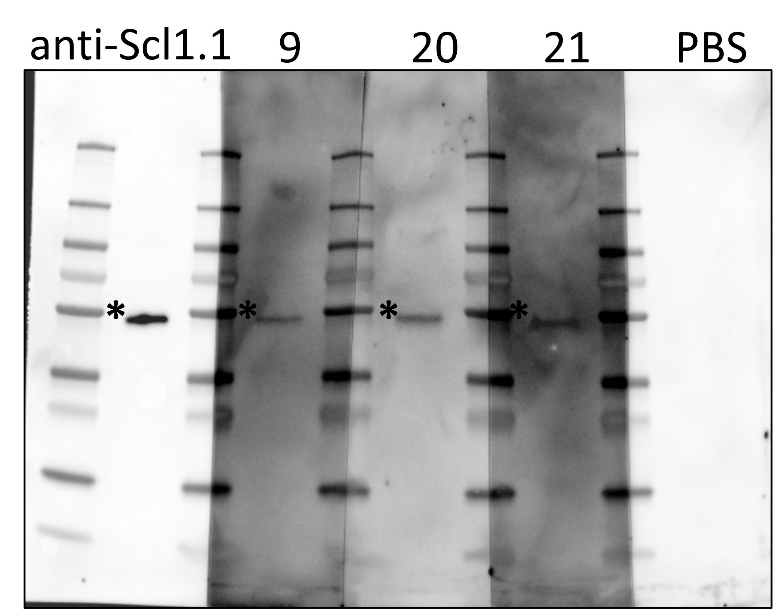
**

**Supplementary Figure 5. Mice weakly seroconvert to M1-GAS Scl1.1 antigen.** (A) Mouse seropositivity toward M1-GAS Scl1.1 antigen developed during tumor colonization via ELISA. Mouse sera are from M1 wild-type-treated mice (gray) and PBS (black) are numbered as in figure 3D. PBS mice represent the average of 5 PBS-treated mice. Dashed red line indicates the average of all 5 PBS mice and is used as a baseline-reactivity control. Sera (1:100 dil.) were tested for immunodetection in wells immobilized with rScl1.1 protein. Each bar represents three technical replicates from individual mice plus SD. (B) Mouse seroconversion to Scl1.1 antigen by western immunoblotting. Sera from A (1:100) were tested against rScl1.1 protein separated by SDS-PAGE and blotted onto a membrane. Asterisks indicate the immunoreactive band for Scl1.1. Numbers correspond to mice from main figure 3D. Anti-Scl1.1-specific Ab is used as positive control in lane 1 and serum from a control mouse injected with PBS is shown in last lane.


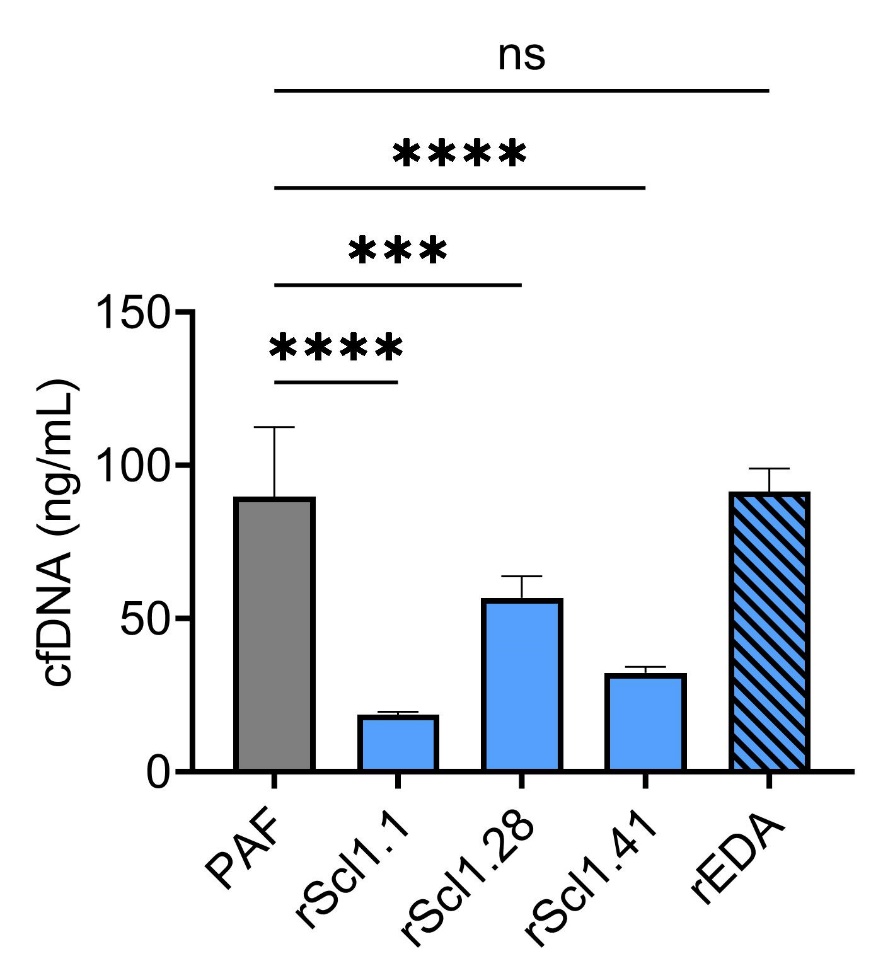


**Supplementary Figure 6. Recombinant Scl1 proteins inhibit NET formation.** Effect of recombinant proteins on NET formation as a quantification cfDNA concentration by Picogreen assay following co-incubation of neutrophils isolated from bone marrow of mice and indicated rScl1 or rEDA control proteins. Significance determined by one-way ANOVA with multiple comparison test. ***=p<0.001, ****=p<0.0001.

**
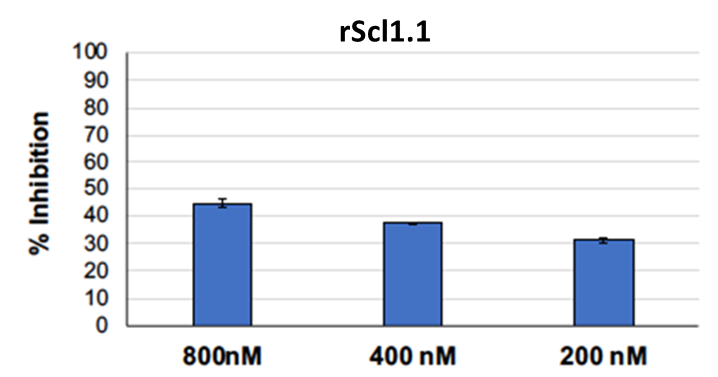
**

**Supplementary Figure 7. Recombinant Scl1.1 inhibits MPO activity.** Effect of rScl1.1 on MPO activity as a percentage of MPO inhibition using a commercially available biochemical screening assay.
